# One-Shot Learning with Pseudo-Labeling for Cattle Video Segmentation in Smart Livestock Farming

**DOI:** 10.3390/ani12050558

**Published:** 2022-02-23

**Authors:** Yongliang Qiao, Tengfei Xue, He Kong, Cameron Clark, Sabrina Lomax, Khalid Rafique, Salah Sukkarieh

**Affiliations:** 1 Australian Centre for Field Robotics (ACFR), Faculty of Engineering, The University of Sydney, Sydney, NSW 2006, Australia; khalid.rafique@sydney.edu.au (K.R.); salah.sukkarieh@sydney.edu.au (S.S.); 2 School of Computer Science, Faculty of Engineering, The University of Sydney, Sydney, NSW 2006, Australia; txue4133@uni.sydney.edu.au; 3 Department of Mechanical and Energy Engineering, Southern University of Science and Technology, Shenzhen 518055, China; he.kong@sydney.edu.au; 4 Livestock Production and Welfare Group, School of Life and Environmental Sciences, Faculty of Science, The University of Sydney, Sydney, NSW 2006, Australia; cameron.clark@sydney.edu.au (C.C.); sabrina.lomax@sydney.edu.au (S.L.)

**Keywords:** one-shot learning, video segmentation, pseudo-labeling, precision livestock farming, deep learning

## Abstract

**Simple Summary:**

Deep learning-based segmentation methods rely on large-scale pixel-labeled datasets to achieve good performance. However, it is resource-costly to label animal images due to their irregular contours and changing postures. To keep a balance between segmentation accuracy and speed using limited label data, we propose a one-shot learning-based approach with pseudo-labeling to segment animals in videos, relying on only one labeled frame. Experiments were conducted on a challenging feedlot cattle video dataset acquired by the authors, and the results show that the proposed method outperformed state-of-the-art methods such as one-shot video object segmentation (OSVOS) and one-shot modulation network (OSMN). Our proposed one-shot learning with pseudo-labeling reduces the reliance on labeled data and could serve as an enabling component for smart farming-related applications.

**Abstract:**

Computer vision-based technologies play a key role in precision livestock farming, and video-based analysis approaches have been advocated as useful tools for automatic animal monitoring, behavior analysis, and efficient welfare measurement management. Accurately and efficiently segmenting animals’ contours from their backgrounds is a prerequisite for vision-based technologies. Deep learning-based segmentation methods have shown good performance through training models on a large amount of pixel-labeled images. However, it is challenging and time-consuming to label animal images due to their irregular contours and changing postures. In order to reduce the reliance on the number of labeled images, one-shot learning with a pseudo-labeling approach is proposed using only one labeled image frame to segment animals in videos. The proposed approach is mainly comprised of an Xception-based Fully Convolutional Neural Network (Xception-FCN) module and a pseudo-labeling (PL) module. Xception-FCN utilizes depth-wise separable convolutions to learn different-level visual features and localize dense prediction based on the one single labeled frame. Then, PL leverages the segmentation results of the Xception-FCN model to fine-tune the model, leading to performance boosts in cattle video segmentation. Systematic experiments were conducted on a challenging feedlot cattle video dataset acquired by the authors, and the proposed approach achieved a mean intersection-over-union score of 88.7% and a contour accuracy of 80.8%, outperforming state-of-the-art methods (OSVOS and OSMN). Our proposed one-shot learning approach could serve as an enabling component for livestock farming-related segmentation and detection applications.

## 1. Introduction

The demand for livestock production, as the second largest human food supplier, is rapidly rising as a result of the growth in population and incomes and changes in lifestyles and dietary habits [1,2]. Cattle farming has become one major commodity in the livestock industrial section. In order to improve the production yield (e.g., meat, milk), significant intensification in livestock farming has occurred, achieved by increasing animal densities and production units [3]. Industrial-scale livestock production is the most common and widespread means of livestock production, which relies on sensors, big data, and machine learning technologies to improve management efficiency, reduce production costs, and enhance animal welfare [4]. Individual animal information acquisition and analysis is desired for “per-animal” production management and increasing profits.

Farmers that acquire accurate information on each animal can monitor their welfare and growth during the whole life cycle of individual cattle, and make the optimal feedlot management strategies [5]. As a non-contact approach, vision-based monitoring has been attracting growing interest from both academia and industry. Cattle video contains valuable motion and behavior information, and the extracted visual features (e.g., appearance phenotype, motion parameters, pose and behavior patterns) can be used for evaluating cattle health and welfare such as lameness detection, live weight prediction, body condition score evaluation and behavior recognition [6,7]. For this, segmenting the animal from the background and obtaining their accurate body contour is a prerequisite. However, traditional segmentation methods based on optical flow, graph partitioning or frame difference struggle to accurately segment the animal in complicated outdoor environments.

Convolutional Neural Network (CNN)-based approaches with strong feature learning ability have achieved great success in segmentation tasks [8]. Since fully convolutional network (FCN)-based semantic segmentation was proposed by [8] in 2015, a variety of deep learning-based approaches have been developed for image segmentation [9]. He et al. [10] proposed Multi-Scale and Pyramid Network-Based models that merge low and high-resolution features to enhance segmentation performance. Qiao et al. [11] implemented the Mask R-CNN with image enhancement for beef cattle image segmentation. The majority of the above deep learning-based approaches need to train models on at least thousands of pixel-level labeled images to guarantee segmentation performance.

However, in the field of livestock farming, the amount of available public data is scarce [12]. Moreover, producing pixel-wise labels for images acquired from a feedlot or paddock is time-consuming (taking at least several minutes) as cattle have an irregular body contour, and their body posture changes with movement. In addition, in real farming management, motion variation in the videos and the influence of changing illumination, shadows and complex backgrounds (e.g., farm facilities, crush or wet ground) will also lead to the degradation of the segmentation performance.

In recent years, to reduce the reliance on large-scale labeled data, one-shot learning and pseudo-labeling approaches have been introduced to handle many visual tasks such as image classification and video segmentation [13,14]. On the one hand, one-shot learning methods utilize one labeled sample and prior knowledge (e.g., data, model and algorithm) to generalize the deep learning networks to new tasks. Two influential one-shot video segmentation methods are OSVOS [15] and OSMN [13]. OSVOS [15] adopted the FCN architecture to transfer generic semantic information to the foreground segmentation task, and then learn the appearance of annotated objects of the test sequence. OSMN [13] utilized the one labeled frame and the previous frame’s spatial information to improve the segmentation performance.

Alternatively, pseudo-labeling (PL) is a typical technique in leveraging unlabeled data by alternating the pseudo-label prediction and feature learning, which improves the model prediction performance for unlabeled data. For example, Tokunaga et al. [14] utilized pseudo-labeling and class proportion to realize semantic segmentation. Ohkawa et al. [16] proposed consensus pseudo-labeling for segmenting the hand image. Zou et al. [17] generated structured pseudo-labels for semantic segmentation.

Inspired by the above, in this study, in order to balance the segmentation accuracy and speed with one labeled image in complex background environments, one-shot learning with a PL-based cattle video segmentation approach is proposed for smart livestock farming. Our proposed approach includes two main parts: Xception-based Fully Convolutional Neural Network (Xception-FCN) and PL. More specifically, Xception-FCN is responsible for feature extraction, and it localizes dense prediction based on CNN feature fusion; the PL leverages pseudo-labels (i.e., the segmented results of the Xception-FCN model on unlabeled data) to re-train the model that leads to boosts in performance on cattle video segmentation.

The main contributions of this study are summarized as follows: (1) we propose a framework of a one-labeled frame-based animal video segmentation approach for precision livestock farming, which takes advantage of both one-shot learning and pseudo-labeling to enhance the segmentation performance. In particular, the proposed Xception-FCN extracts CNN features using depth-wise separable convolutions, and then fuses these CNN features to increase representational efficiency and reduce over-fitting probability; the pseudo-labeled data generated from the Xception-FCN model were then utilized to boost the segmentation performance; (2) comprehensive comparative experiments were conducted on a real acquired cattle video dataset to validate the effectiveness of the proposed approach. The proposed approach achieved 88.7% mIoU and 80.8% contour accuracy, which outperformed the state-of-the-art methods (e.g., OSVOS and OSMN); (3) the effects of pseudo-labeling and pre-training on segmentation performance were also investigated. Experimental results show that both pseudo-labeling and pre-training can enhance the segmentation performance. Our proposed approach achieved accurate and real-time cattle video segmentation using only one labeled image frame, and could serve as an enabling component of a comprehensive solution to the automatic animal appearance phenotype and monitoring in the field of smart livestock farming.

The remainder of this paper is organized as follows: Section 2 briefly reviews the related works of one-shot learning, pseudo-labeling and video segmentation; Section 3 describes our proposed video segmentation method; Section 4 introduces the experimental dataset, network parameters, and evaluation methods; Section 5 presents cattle video segmentation results; discussions of the performance are presented in Section 6; finally, conclusions are given in Section 7.

## 2. Related Work

### 2.1. One-Shot Learning

In deep learning, one-shot learning [18] that uses only one labeled training image has been increasingly attracting more attention. One-shot learning methods have model generalization ability, which could be easily transferred to different tasks based on three aspects of prior knowledge—data, model and algorithm [19]. In terms of the data aspect, these methods use prior knowledge to augment the training set and enlarge the size of the training samples. Kwitt et al. [20] explored how the transient classes are represented in the feature space and how the new data can be transferred from the existing data. Douze et al. [21] proposed a semi-supervised method based on label propagation to leverage the effects of a large collection of images. Wu et al. [22] utilized the pseudo-labeled tracks to gradually update the person re-identification model.

Furthermore, model prior knowledge can share parameters for different learning tasks. For example, two natural language processing tasks about legal texts can be dealt with together to simultaneously infer the attributes and charges [23]. The auto-encoder can first be pre-trained on source tasks to obtain the generic information and then applied to the target task [24]. The transfer between models can also be applied in an embedding space [25]. With regard to the algorithm transfer, refining existing parameters is commonly used in the fine-tuning process. Yoo et al. [26] refined the parameters in a group-wise way by clustering them using the pre-trained filters on CNN, and pre-trained functions from unlabeled data were used to cluster and separate samples in [27].

### 2.2. Pseudo Labeling

The main principle of PL is to leverage unlabeled data by alternating the pseudo-label prediction and feature learning [28]. This firstly trains a model using the limited labeled data, the trained model is then applied to the unlabeled data to generate pseudo-labels and these pseudo-labels are used to re-train the model for boosting the final performance [29]. Through this simple and effective self-training method, PL improves the network performance using limited labeled data. For example, Lee et al. [30] exploited the trained model to generate pseudo-labels for the unlabeled data, and then fine-tuned the model to fully leverage the unlabeled data.

Recently, Pan et al. [28] utilized the pseudo-label learning to minimize the distribution of target-domain data with the source-domain prototypes. For unsupervised semantic segmentation, Zou et al. [31] introduced a pseudo-label strategy to the semantic segmentation and provided one comprehensive analysis of the regularization terms. Most recently, Zheng and Yang [32] applied the pseudo-labels to learn the domain-specific features, yielding competitive results. Sindagi and Patel [33] leveraged the scene-wise pseudo-labels to transfer the trained model to a new task. In short, PL can assist unlabeled data in their supervision of the training of the model. All the above research has illustrated that PL has the capability to improve deep learning network performance with only a limited amount of labeled data [29].

### 2.3. Video Segmentation

Video segmentation refers to analyzing video frames and segmenting them into regions of interest. According to the required level of supervision, video segmentation techniques can be broadly categorized into unsupervised, semi-supervised and supervised methods.

- Unsupervised segmentation can be achieved by motion analysis, trajectory clustering, or object proposal ranking [34]. Faktor and Irani [35] found motion salient regions by extracting dominant motion for video object segmentation. Xiao and Jae Lee [34] generated a set of spatio-temporal bounding box proposals, and then refined them to obtain pixel-wise segmentation proposals. Recently, Wang et al. [36] proposed unsupervised video object segmentation through visual attention. Li et al. [37] transferred the knowledge encapsulated in image-based instance embedding networks for unsupervised video object segmentation. Although unsupervised approaches do not rely on data labeling, the underlying segmentation hypotheses restricted their applications in high-complexity datasets.
- Semi-supervised segmentation propagates the label information of candidate objects in one or a few key-frames to all video frames [15,38]. Tsai and Huang [39] incorporated motion analysis and image processing techniques in video sequences for the automatic detection of cattle behavior. Liu et al. [40] trained a classifier on low-level hand-crafted features with limited data to process videos for farming automation. Deep learning approaches such as OSMN [13] and FEELVOS [41] began to utilize motion and spatial relationships without fine-tuning. Ventura et al. [42] proposed a Recurrent Neural Network (RNN)-based approach to utilize the temporal correlation between video frames. Feng et al. [43] classified the complex agricultural planting structure with a semi-supervised extreme learning machine framework. Semi-supervised segmentation reduces the need for large labeled datasets but still requires many iterations of optimization for real-world applications.
- Supervised segmentation requires tedious user interaction and iterative human corrections [44], which can attain high-quality boundaries and is more favorable for specific scenarios such as video post-production. Tokmakov et al. [45] combined the outputs of pre-trained appearance and a motion network to generate final segmentation results. Similarly, Xu et al. [46] proposed a sequence-to-sequence model that learns to generate segmentations from sequences of frames. Unfortunately, these performance achievements rely on large amounts of labeled training data, and data labeling is expensive and time-consuming.

For video analytics applications in precision agriculture and livestock farming, Milioto et al. [47] achieved the real-time semantic segmentation on crop and weed based on the encoder–decoder architecture network. Zhang et al. [48] estimated animal poses from video data. The cow joints’ spatial positions were analyzed by [49] with two CNN structures and a post-processing module.

Through the strong learning capability of the neural networks and the availability of large-scale pixel-label datasets, the video object/animal segmentation task was formulated as a one-shot problem [15]. The one-shot learning approach usually trains an offline model to produce an initial estimation, and then fine-tunes the model using the available ground-truth. For animal video segmentation tasks, to avoid the high-cost of data labeling, one-shot learning with PL is more suitable as it only needs to label one image-frame [50].

## 3. The Proposed Approach

### 3.1. Overview of the Proposed Approach

**Figure 1:**
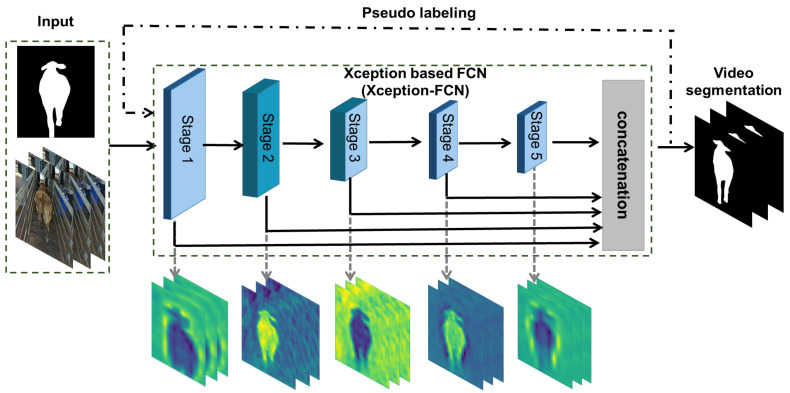
The framework of one-shot learning with PL for cattle video segmentation.

The proposed approach (Figure 1) consists of two main parts: Xception-FCN and PL. Firstly, the input video and its first labeled frame are fed to the pre-trained Xception-FCN for network fine-tuning and initial segmentation result generation. Then, these segmented images are regarded as pseudo-labels to re-train the models and refine the segmentation results. By this, PL filters the outliers from the segmented images and refines animal contours, which enhance the final cattle video segmentation performance.

For a given video *F* with *n* frames (*$f_{1}$, $f_{2}$, ⋯, $f_{n}$*), its first frame $f_{1}$ with the corresponding manually labeled mask $s_{gt}$ and video *F* are input into Xception-FCN for network fine-tuning. Here, the pre-trained weights of Xception-FCN are obtained from public datasets (e.g., PASCAL VOC 2012). After Xception-FCN has completed the fine-tuning process with the only one labeled frame $s_{gt}$, the obtained network model *M* is applied to the unlabeled video frames. The corresponding segmentation results generated from the unlabeled video frames are denoted as *Y* = [*$y_{2}$, ⋯, $y_{n}$*]. Here, *Y* can be regarded as pseudo-labeled data. Then, the labeled image $s_{gt}$ and pseudo-labeled data *Y* are used to re-train the model *M*. The re-trained model *M* is fine-tuned again with the only label frame $s_{gt}$, and then applied to unlabeled video frames to produce more accurate segmentation results $Y_{f}$ = [*$y_{f2}$, ⋯, $y_{fn}$*]. More details of our proposed approach are demonstrated below.

### 3.2. Xception-FCN Architecture

Feature extraction plays an important role in visual recognition and segmentation. VGG and Xception networks have shown good feature representation in vision tasks [51]. However, they are time-consuming due to their lower learning efficiency and large amount of network parameters.

In this work, we improved the Xception network and developed a lightweight Xception-FCN network architecture to extract visual features for cattle video segmentation. According to our experiments with different layer-length backbones, an Xception architecture with 20 convolutional layers was confirmed as an Xception-FCN backbone. Compared with the original Xception (65 layers), the proposed Xception-FCN significantly reduces the training parameters but retains the network performance.

**Figure 2:**
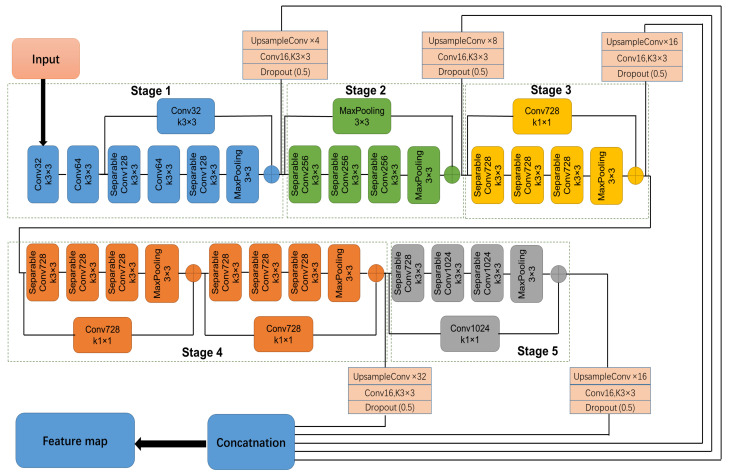
The proposed Xception-FCN network architecture.

As illustrated in Figure 2, there are five main blocks in Xception-FCN, and between the different blocks there is a MaxPooling layer that down-samples feature maps. In each block, the dilated convolution with different dilation rates is used to expand the receptive field. Xception-FCN extracts CNN features from five different layers, and then fuses them for final video segmentation. The features obtained from the first three blocks mainly contain spatial information such as edge, texture and shape, whilst the features extracted from the last two blocks have more semantic information.

Given one video frame *f* and learned weight *w*, the process of extracting features can be represented by

(1) $$X_{n}=l_{n}\left(\left(\cdots l_{2}\left(l_{1}\left(f;w_{1}\right);w_{2}\right)\cdots \right);w_{n}\right)$$

where $l_{1}$, $l_{2}$, ·, $l_{n}$ are activated functions at different layers, and $X_{n}$ is the extracted feature map at the *n*-th layer.

In general, with the layer going deeper, the extracted features from Xception-FCN are gradually transferring from spatial to semantic and abstract information. How to leverage different layer features for segmenting the cattle from video is challenging. Based on our experimental testing, the feature maps from five different stages were chosen and concatenated for segmenting cattle video. These utilized five feature maps containing the shallow layers’ low-level and deep layers’ high-level visual features that enhance the cattle representation ability of video segmentation.

As these five feature maps’ sizes are different, we first upsample them to make their sizes the same as that of the video frame. Then, these upsampled features were concatenated together to generate the final feature $X_{final}$:

(2) $$X_{final}=Up{\left\{X_{5}\right\}}_{↑4}++Up{\left\{X_{8}\right\}}_{↑8}++Up{\left\{X_{11}\right\}}_{↑16}++Up{\left\{X_{17}\right\}}_{↑16}++Up{\left\{X_{20}\right\}}_{↑32}$$

where $X_{5},\cdots ,X_{20}$ are visual features from the corresponding layer number; Up{} indicates the upsampling operation, and the subscripts ↑4,↑8,↑16,↑32 indicate upsampling ratios with 4, 8, 16 and 32, respectively.

By inputting the one labeled frame to the Xception-FCN network, a fine-tuned model *M* is obtained. This process is regarded as a one-shot learning process. After applying *M* to the unlabeled video sequence (*$f_{2}$, ⋯, $f_{n}$*), the initial segmentation results *Y* = (*$y_{2}$, ⋯, $y_{n}$*) are obtained.

### 3.3. Pseudo-Labeling for Cattle Video Segmentation

Segmenting the animal from its background in a video with only one labeled image (foreground/background information in this frame) is challenging. Although Xception-FCN can segment cattle from the background using one-shot learning, there are some false segmentation problems such as noise and contour errors. In order to reduce the segmentation errors and leverage the unlabeled data information, pseudo-labeling was considered to re-train the model and boost the segmentation performance.

The unlabeled data’s segmentation results (obtained from Xception-FCN) *Y* = (*$y_{2}$, ⋯, $y_{n}$*) and the first labeled frame $s_{gt}$ are combined and used to re-train the Xception-FCN model *M*. The re-trained model *M* is again fine-tuned with the only label frame $s_{gt}$, and then applied to segment the cattle video, and output the final obtained segmentation results $Y_{f}$ = (*$y_{f2}$, ⋯, $y_{fn}$*). By this, we yield large gains in segmentation accuracy without extra additional labeled data or inference cost.

## 4. Experimental Setup

### 4.1. Datasets

To verify the effectiveness of the proposed video segmentation approach, extensive experiments were conducted on our cattle video dataset acquired from a Southern Queensland commercial feedlot. Cattle videos were recorded when the cattle were walking along the crush race (path). Rear-view videos were recorded using the ZED camera in three different months during the feed period (i.e., induction, middle and end point) on the 20 March, 30 April and 30 May 2018, respectively. The animal study was reviewed and approved by University of Sydney Animal Ethics Committees (AEC).

**Figure 3:**
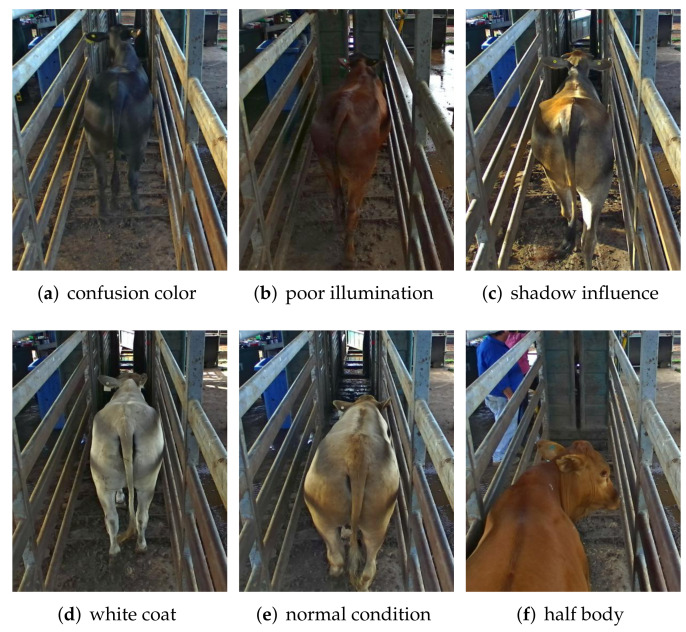
Samples of images in the challenging cattle dataset.

A total of 22 cattle videos (i.e., 809 frames) were selected for our experiments (each video was acquired from a different cattle), and the video length varied from 16 frames to 68 frames due to the different cattle walking speeds. Each video begins with the cattle walking into the crush (from the moment that the cattle body trunk can be seen), and the video ends when the cattle hips leave the exit gate. As illustrated in Figure 3, this dataset is challenging due for the following reasons: (1) the cattle frequently changed body posture and moved fast when they were driven into the cattle crush; (2) the cattle coat colors were different, and the color of some cattle had a high similarity with the soil background; (3) the cattle shape becomes small and unclear at the end of the fence, which are even difficult to distinguish for human beings; (4) the lighting condition changes during the day time. Different illumination and shadows appeared in the video images. These lighting issues require the deep learning model to have a strong generalization ability.

### 4.2. Network Pre-Training and Fine-Tuning Details

Our experiments were implemented using Python and PyTorch on a computer equipped with NVIDIA RTX 2080Ti GPU and Ryzen 5 3600 3.6 GHz CPU. The implementation of our proposed approach mainly includes pre-training, fine-tuning and re-training steps.

- Network pre-training: Several open datasets were used to pre-train the proposed Xception-FCN network, and based on the datasets used, we classified the pre-training process into base and objectness training. For base training, a total of 11,840 images from the PASCAL VOC 2012 dataset [52] and an extended dataset [53] were used. In terms of objectness training, the DAVIS 2016 dataset containing 30 videos (not including cattle videos) was used.
  For the pre-training, the optimization algorithm used was stochastic gradient descent (SGD) and the whole process had 45,000 iterations (25,000 iterations for base training and 20,000 iterations for objectness training). During the pre-training process, the used learning rate gradually declined from $10^{-6}$ to $2.5\times 10^{-7}$. With the help of pre-training weights, the optimized Xception-FCN network has the certain capability of segmenting foreground objects (i.e., cattle) from the video.
- Network fine-tuning: After pre-training, the manually labeled one image (first frame) of the testing video was used to fine-tune the proposed Xception-FCN network. To maximize the effectiveness of this one labeled image, some typical data augmentation techniques such as flipping, cropping, brightness, zooming and contrast change were also used. For network fine-tuning, the learning rate was set to $10^{-7}$. As the light-weight Xception-FCN architecture and separable convolution utilized our approach, the model efficiency of the video segmentation was enhanced. Additionally, based on the experimental comparison, optimized fine-tuning iterations were set to 100 in consideration of the speed and accuracy of the cattle video segmentation.
- Network re-training: for further reducing the segmentation noises and contour errors, pseudo-labels, namely the initial segmentation results generated by Xception-FCN, combined with the one labeled frame, were used to re-train the model. The re-trained epochs were set to 100 and the learning rate was set to $10^{-7}$. The other parameters were the same as those used in the process of network fine-tuning.

### 4.3. Performance Evaluation

In order to evaluate the video segmentation performance, four popular measure metrics—region similarity regarding intersection over union J, contour accuracy F, the temporal instability of the masks T, and testing speed (second per frame) were used in our experiments. The metric J measures the matching of ground truth and prediction, while F reflects the contour accuracy. The metric T estimates the deformation among the segmented frames, where high temporal instability means strong occlusions and deformations.

For segmentation output *Y* and the corresponding ground-truth mask *G*, J can be computed by

(3) $$J=\frac{\left|Y\cap G\right|}{\left|Y\cup G\right|}$$

Contour accuracy F can be computed using the contour based precision $P_{c}$ and recall $R_{c}$:

(4) $$F=\frac{2P_{c}R_{c}}{P_{c}+R_{c}}$$

In our experiments, both J and F have three different values: mean (the average results of all segmented video frames), recall (the average results for the frames with a large threshold of 0.5) and decay (performance loss over time, which was computed using the segmentation result of the first frame minus the last frame [54]). Higher values of J and F indicate good segmentation performance, while higher decay value represents poor video segmentation.

## 5. Experimental Results

### 5.1. Comparison of Different Segmentation Methods

To verify the effectiveness of the proposed approach, the real acquired cattle videos in a feedlot were used for verification and a comparison was carried out with state-of-the-art methods, namely OSVOS and OSMN. Here, OSVOS and OSMN were implemented on our cattle dataset using their public codes and weights to compare with our proposed approach. Table 1 illustrated the video segmentation results of different methods.

As shown in Table 1, our proposed one-shot learning-based approach achieved a J(Mean) of 88.7%, J(Recall) of 99.8%, F(Mean) of 80.8%, and F(Recall) of 97.7% on cattle video segmentation. These achieved values are significantly higher than that of OSVOS (84.4% J(Mean), 75.0% F(Mean)) and OSMN (80.0% J(Mean), 62.1% F(Mean)). Meanwhile, the temporal instability T of our proposed approach is 45.2%, which is lower than that of OSVOS (46.2%) and OSMN (47.4%). The J(Mean) and F(Mean) of our approach are significantly higher than those of OSVOS and OSMN. These improvements illustrate that our proposed approach is favorable for high-accuracy video segmentation tasks in smart livestock farming.

The ablated version without PL module also achieved 87.6% J(Mean) on the cattle dataset, which demonstrated that our Xception-FCN network architecture is more advanced for segmenting animals. Overall, the proposed one-shot learning with the pseudo-labeling network architecture has strong feature learning and extraction ability, which can feasible be used for cattle video segmentation in the smart livestock farming applications.

For testing the speed of our approach on cattle video segmentation, the computational efficiency of our proposed approach, OSVOS and OSMN are also given in Table 1. The testing speed of our approach is 0.44 s/frame, which is faster than those of OSVOS and OSMN.

**Figure 4:**
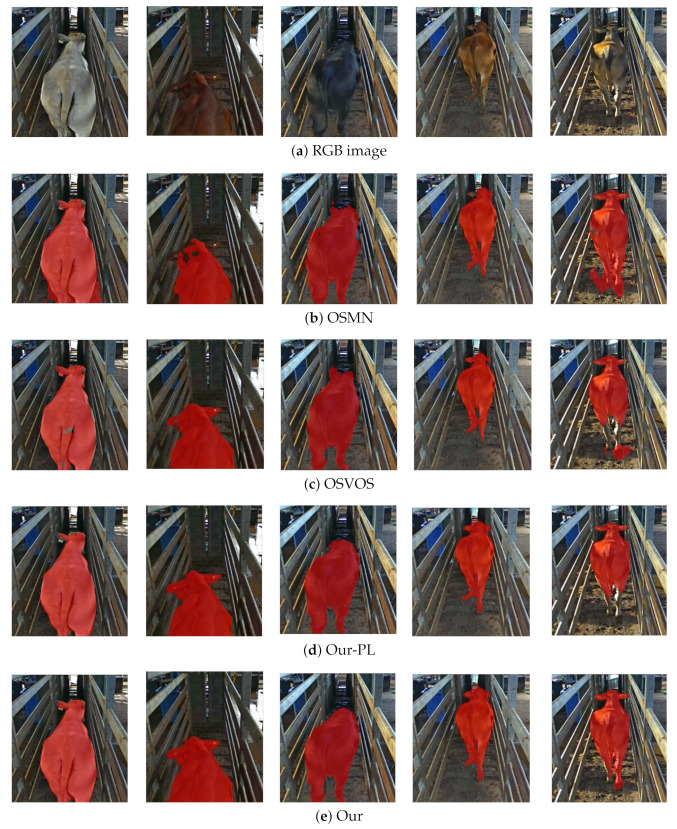
Segmentation results of different approaches on the cattle dataset.

The qualitative comparison of different approaches (i.e., OSMN, OSVOS and our approach) on our cattle dataset is demonstrated in Figure 4. It can be seen that the segmented cattle body area (red region) are obviously under-segmented in OSMN (Figure 4b)—especially the regions nearby the cattle hips—while the tail and legs are missed in OSVOS Figure 4c. However, our proposed approach can segment cattle with a highly accurate cattle contour (Figure 4d), and offers a significantly superior performance to those of OSMN and OSVOS.

The last frame in Figure 4d also illustrates that the proposed approach could significantly reduce the influence of shadow and illumination. In addition, it can be noticed that different cattle coat color such as white, brown and dark shown in Figure 4a are also well segmented (displayed in Figure 4d). The above findings validate the robustness of our approach. The proposed approach could be used for different cattle breeds in complex farm scenes (e.g., different background, changing illumination and varying pose status), which has potential commercial application value in the field of automatic cattle segmentation and monitoring.

### 5.2. Qualitative Analysis

**Figure 5:**
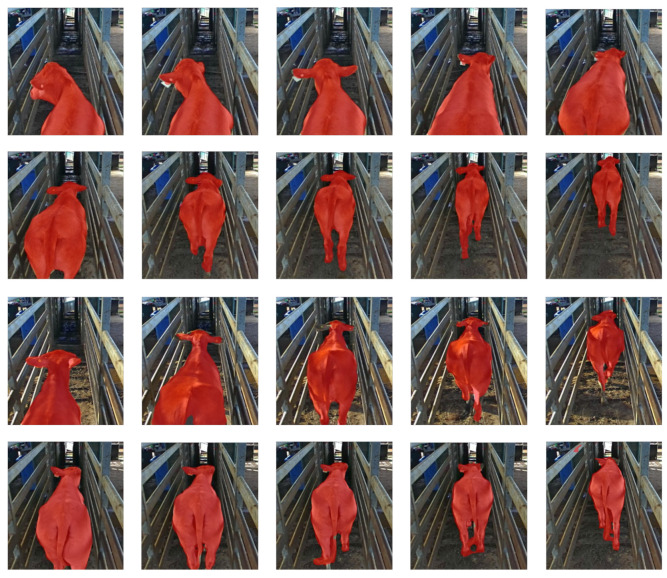
Qualitative results of our approach on the cattle dataset.

More cattle video segmentation examples of the proposed one-shot learning approach are shown in Figure 5. Here, four typical video segmentation examples are displayed. They contain the situations of changing illumination, shadow influence, cattle movement and posture variation. Due to the content restriction, only four image frames in each video are presented, and each row’s images are from the same video. In Figure 5, each animal is walking forward in the crush. It can be seen that the image frames in each video are well segmented, except for a few false segmented tail parts. The cattle body parts, including ears, heads, legs, and body contours, were well segmented and can be clearly seen. In general, the proposed one-shot learning with pseudo-labeling achieved satisfactory cattle video segmentation with only one label image under complex background conditions (e.g., different illuminations, cattle movement and posture changing).

### 5.3. Ablation Study: Effect of PL

To analyze and quantify the significance and effects of pseudo-labeling in our approach, comparison experiments were conducted on ablated versions and the results are illustrated in Table 1.

Our approach without PL obtained 87.6% J(Mean), 98.6% J(Recall), 79.4% F(Mean) and 96.4% F(Recall). However, with the PL module, our approach segmentation performance was significantly improved. Specifically, the J(Mean) increased by 1.1%, J(Recall) increased by 1.2%, while F(Mean) improved by 1.4% and F(Recall) increased by 1.3%. Moreover, with the help of PL, the effective temporal instability of the masks T in the segmented video was also improved by 3.7%, which means the last image frame of the video was also well segmented. These results illustrated that pseudo labels generated from Xception-FCN using only one label frame could help refine the network segmentation performance, especially when a large amount of unlabeled data is available. In terms of running-speed, although the PL module brings an extra 0.02 s time cost, it does boost the segmentation accuracy.

### 5.4. Ablation Study: Effect of Pre-Training

In our work, the influence of the pre-training process on cattle video segmentation was also investigated. As the pre-training process consists of base training (BT) and objectness-training (OT), effects of both BT and OT on video segmentation were therefore analyzed. As illustrated in Table 2, BT and OT contribute 11.6% and 1.3% to J(Mean), respectively. In terms of F(Mean), BT and OT enhance the segmentation performance by 14.1% and 5.2%, respectively. These achievements show that OT makes a greater contribution than BT. One possible reason for this is that OT focuses on pixel objectness using a binary cross-entropy loss, so that the optimized network parameters by OT could significantly reduce false segmentation and contour blur among these segmented images.

Apart from improving the accuracy and time instability, BT and OT also effectively decrease J(Decay) by 19.3% and 8.7%, respectively. This demonstrates that the pre-training modules of BT and OT can improve the performance in accuracy and time instability in general.

## 6. Discussion

Here, we proposed a one-shot learning approach with pseudo-labeling for cattle video segmentation. Unlike the works in [11,55] which used a large amount of labeled data to segment cattle, the proposed one-shot learning approach leverages the unlabeled data to boost the final segmentation performance. Our experimental results demonstrated the proposed approach’s effectiveness for livestock segmentation and monitoring tasks. The proposed approach provides an effective alternative to methods that require a large amount of data-labeling for deep learning-based livestock farming applications.

### 6.1. Influence of the Cattle Phenotypic Appearances on Video Segmentation

Cattle phenotypic appearances such as coat color, body shape and body size are important indicators of animal welfare and farming management. A robust and high-accuracy segmentation method that is not sensitive to cattle phenotypic appearances is vitally needed for smart farming. As Figure 5 shows, cattle with different coat colors (e.g., brown, white, dark) were well segmented by our method. In addition, in our videos acquired from three different months, the corresponding cattle have different body sizes and weights, but the proposed Xception-FCN model could still segment the cattle in videos with 88.7% J(Mean) and 80.8% F(Mean). These results illustrated that cattle phenotypic appearances could not bring much influence on the performance of our proposed approach.

### 6.2. Analysis of the Influence of Motion on Video Segmentation

Videos usually contain temporal cues, especially in situations where the cattle perform repeated behaviors or activities. Our proposed approach labeled the first video frame for cattle segmentation, which is favorable for the situation in which the cattle exhibit normal behaviors. It could be noticed that with cattle walking far away from the camera, their body scale and movement posture are continuously changing, but the overall segmentation performance is favorable except for a few body parts such as hooves and tails. The main reason is that the tiny body parts such as tails and hooves account for a small proportion of the whole body, and few visual features can be extracted from these parts for segmentation.

Our video segmentation approach uses one labeled image (i.e., the first video frame) to segment cattle in the video, reducing the data labeling costs. It should be noted that if abnormal behaviors appeared, the large motion and occlusion in cattle video sequences will lead to unstable segmentation performance. In addition, as discussed in [56], if the first frame has low image quality or does not contain the complete object shape, it will not be the best choice for data labeling. In the future, the best guidance frame selection [56] will be considered. Meanwhile, long-term dependency on video sequences and motion deformation tracking will also be investigated to further enhance the segmentation performance.

### 6.3. Analysis of the Proposed Approach’s Applicability

The proposed approach is a light-weight and effective animal video segmentation framework, which combines one-shot learning and pseudo-labeling to achieve segmentation with one labeled image. As illustrated in Figure 4, the cattle are well segmented under the conditions of changing illumination and shadow influence. In the proposed approach, Xception-FCN fuses low and middle level features, and pseudo-labeling utilizes pseudo-labels (generated by Xception-FCN model) to boost the segmentation performance. Although experiments were only conducted on cattle video data in this paper, the proposed approach also could be applied to other animal species.

On the other hand, data labeling is a prerequisite for deep learning-based vision tasks, especially in the field of precision agriculture and smart livestock farming. Considering the time and costs of data labeling, the proposed approach could have wide applications in real livestock farm management. In addition, this approach can also be applied to assist an unmanned aerial vehicle (UAV) or unmanned ground vehicle (UGV) in locating animals or detecting or analyzing animal behaviors.

## 7. Conclusions

Cattle segmentation is a prerequisite to automatic body parameter measurements and behavior recognition in smart livestock farming. Unlike other deep learning-based approaches which rely on large-scale labeled data, in this study, one-shot learning cattle video segmentation with pseudo-labeling is proposed. In the proposed approach, Xception-FCN utilizes one labeled image and pre-training weights to generalize deep learning to cattle segmentation; then, these segmented results are used as pseudo-labeled data to refine the segmentation results. Extensive experiments were conducted on cattle videos in real farming environments. Experimental results show that the proposed approach obtained the segmentation performance with 88.7% J(Mean) and 80.8% F(Mean), outperforming OSVOS (84.4% J(Mean), 75.0% F(Mean)) and OSMN (80.0% J(Mean), 62.1% F(Mean)). The proposed approach leverages the effects of fine-tuning and unlabeled data to enhance visual feature representation ability, which boost the performance. Moreover, even without the PL module, our approach still achieved 87.6% J(Mean) and 79.4% F(Mean), which is also significantly higher than the values of OSVOS and OSMN. This result demonstrated that the proposed Xception-FCN is an effective deep learning architecture and is favorable for video segmentation tasks. Overall, the proposed approach achieved a fast and highly accurate one-shot video segmentation, which is favorable for the applications of animal segmentation and monitoring in smart livestock farming.

## Figures and Tables

**Table 1:** Comparison of the different video segmentation methods.

	J(M)↑	J(R)↑	J(D)↓	F(M)↑	F(R)↑	J(D)↓	T↓	Time (s/f)
OSMN	80.0	93.4	16.6	62.1	74.6	11.3	47.4	1.21
OSVOS	84.4	97.5	13.9	75.0	89.4	14.7	46.2	0.76
Ours-PL	87.6	98.6	10.6	79.4	96.4	12.3	48.9	0.42
Ours	88.7	99.8	9.0	80.8	97.7	10.7	45.2	0.44

“M” is short for mean, “R” represents recall and “D” indicates decay. Noticeably, the up-arrows beside the metrics indicate that the higher the metric is, the better it is. Similarly, the down-arrows indicate that a lower figure is preferred. Note that since the original OSMN does not contain fine-tuning, for a fair comparison, fine-tuning was added to the used OSMN.

**Table 2:** Comparison of our approach against the downgraded version without pre-training.

	J(M)↑	J(R)↑	J(D)↓	F(M)↑	F(R)↑	J(D)↓	T↓
Ours	88.7	99.8	9.0	80.8	97.7	10.7	45.2
Ours-BT	77.1	82.4	30.2	66.7	73.0	30.0	56.5
Ours-OT	87.4	99.8	10.9	75.6	92.4	19.4	41.1

## Data Availability

All the generated data will be available upon reasonable request.
